# ACD: An Adaptable Approach for RFID Cloning Attack Detection

**DOI:** 10.3390/s20082378

**Published:** 2020-04-22

**Authors:** Weiqing Huang, Yanfang Zhang, Yue Feng

**Affiliations:** 1 School of Computer and Information Technology, Beijing Jiaotong University, Beijing 100044, China; huangweiqing@iie.ac.cn; 2 Institute of Information Engineering, Chinese Academy of Sciences, Beijing 100093, China; zhangyanfang@iie.ac.cn; 3 School of Cyber Security, University of Chinese Academy of Sciences, Beijing 100093, China

**Keywords:** radio frequency identification (RFID), cloning detection, Floyd-Warshall algorithm

## Abstract

With the rapid development of the internet of things, radio frequency identification (RFID) technology plays an important role in various fields. However, RFID systems are vulnerable to cloning attacks. This is the fabrication of one or more replicas of a genuine tag, which behave exactly as a genuine tag and fool the reader to gain legal authorization, leading to potential financial loss or reputation damage. Many advanced solutions have been proposed to combat cloning attacks, but they require extra hardware resources, or they cannot detect a clone tag in time. In this article, we make a fresh attempt to counterattack tag cloning based on spatiotemporal collisions. We propose adaptable clone detection (ACD), which can intuitively and accurately display the positions of abnormal tags in real time. It uses commercial off-the-shelf (COTS) RFID devices without extra hardware resources. We evaluate its performance in practice, and the results confirm its success at detecting cloning attacks. The average accuracy can reach 98.7%, and the recall rate can reach 96%. Extensive experiments show that it can adapt to a variety of RFID application scenarios.

## 1. Introduction

Radio frequency identification (RFID) is a non-contact automatic identification technology that uses the spatial coupling of radio frequency signals or the transmission characteristics of radar reflections to achieve automatic identification. As a key technology of the internet of things, RFID is widely used in target tracking, access control, automatic payment, indoor positioning, and supply chain management [1,2,3,4,5]. Its widespread application has led the society to increasingly depend on it, with correspondingly high security requirements. However, due to limited on-chip resources (only thousands of logic gates), most low-cost passive tags cannot support sophisticated cryptographic schemes, which makes communication between the reader and the tags vulnerable to security attacks. We focus on the most prevalent security attack, tag cloning.

A clone attack will threaten RFID-assisted applications that use genuineness of valid tags to determine the authenticity of tagged objects, and it can bring huge economic loss and leakage of sensitive information. For example, the pharmaceutical industry has proposed RFID to track drugs through supply chains. Clone tags here may allow counterfeiters to bypass security checks to introduce counterfeit drugs. RFID tags are used in several countries’ electronic passport (ePassport) schemes to prevent forgery. Clone tags may allow terrorists or illegal immigrants to enter a country undetected. Some government departments use RFID tags for access control. Clone tags may allow criminals to enter exhibition halls at will to steal important exhibits. In this type of application, tags are read very frequently. We focus on detection cloning attacks in this type of application.

A clone attack refers to the copying of information of an RFID electronic tag or smart card to a clone tag, which will then have the same characteristics as the original tag, and can replace it. Clone attacks use sniffing, eavesdropping, and other technologies to obtain all the data of the original tag, including encoding and user data, write all of the information to an RFID tag that can write to the entire area, and replicate the tag. Therefore, clone attacks require sniffing, eavesdropping, and reading devices (such as Proxmark III, which is a common tool on the market [6]) and RFID tags that can modify the data (such as the UID-changeable RFID card, also known as the Chinese magic card. UID means user identification). RFID technology is classified by frequency as low frequency (LF), high frequency (HF), ultra-high frequency (UHF), and microwave (UW). Frequency bands have protocols that require different methods of attack. In 2005, Johns Hopkins University and RSA Labs disclosed a cracking method and clone attack process on a digital signature transponder (DST) [7], a type of LF RFID device. Such a cloning attack can realize the cloning of DTS car keys. In 2008, the Dutch government announced the cracking of NXP’s MIFARE Classic RFID chip [8]. Researchers analyzed the chip’s security vulnerabilities and executed a cloning attack by eavesdropping on the communication between the tag and reader. That same year, a Korean laboratory proved the risk of cloning attacks on IC cards. After cloning a financial IC card, a clone tag could carry out illegal financial transactions. RFID cloning attacks [9] have subsequently succeeded in areas, such as public transportation and access control systems, realizing huge economic losses, and negative social impacts.

Aiming at the risk of cloning attacks faced by RFID technology, how to quickly and effectively detect clone tags has attracted much attention. Current research mainly concerns physical layer radio frequency fingerprints [10,11,12], authentication protocols [13,14], key synchronization [15,16,17,18], and tag trajectory analysis [19,20,21]. The physical layer-based RF fingerprint needs to use other equipment for information collection, and the physical layer RF information of the RFID tag is obtained, which cannot be detected in real time. The authentication protocol needs to modify the existing protocol. Currently, physically unclonable functions (PUFs) are widely used in authentication protocols. PUFs are the result of the manufacturing process of integrated circuits (ICs), which introduces random physical variations into the microstructure of an IC, making it unique. Gope et al. proposed a lightweight and privacy-preserving two-factor authentication scheme and a lightweight and practical anonymous authentication protocol using PUFs [22,23]. They are secure, efficient, practical, and effective for the resource-constraint RFID tag. PUF needs special reader and tag, which are relatively expensive to repurchase. In different scenarios and RFID application systems, people will generally use COTS RFID devices at present due to the limit of capital. For this application scenario, the security of the system should also be taken seriously. A synchronous key mechanism requires the tag to have a certain computing capability, which increases the cost of the RFID tag. Tag trajectory analysis is based on existing RFID technology without the increase of cost, and through the spatiotemporal correlation analysis of tag position and trajectory, the detection of clones is performed in a probabilistic or deterministic manner. RFID technology has been widely used in the fields of supply chains, logistics, and transportation. Redesigned tags or protocols are costly, so research based on tag trajectory analysis has received widespread attention.

According to the spatiotemporal correlation analysis of tag trajectory, we propose an adaptable clone detection (ACD) approach based on the Floyd-Warshall algorithm and spatiotemporal collisions. We build a spatiotemporal correlation matrix between adjacent nodes based on the data collection and analysis of an RFID system, and use the Floyd-Warshall shortest path algorithm to model and learn to construct a spatiotemporal correlation matrix between arbitrary nodes. We detect abnormal tags through spatiotemporal collisions in real-time trajectories. To put it simply, a spatiotemporal collision is the appearance of a tag in two distant places within a short time. We have improved the Floyd-Warshall algorithm, and experiments confirm its better real-time performance.

We tested in a work area of about 500 square meters. The results show that the accuracy rate of ACD in a short time can reach 100%, the average accuracy rate can reach 98.7%, and the recall rate can reach 96%. We also conducted experiments in a large venue. We tested the method on about 400,000 data points collected from a product exchange conference and obtained similar detection accuracy. These results indicate that ACD can be deployed to any RFID application in which tags are scanned frequently.

The main contributions of this article are as follows:

1. Utilizing the spatiotemporal collision in spatiotemporal correlations, we propose a clone tag detection method, ACD, based on an improved version of the Floyd-Warshall algorithm. This method can establish the spatiotemporal relationship between nodes when the trajectory is incomplete, and reduce the number of training samples. The improved Floyd-Warshall algorithm can significantly reduce the detection delay.
2. ACD has good applicability and portability. There is no need to add equipment for different types of RFID commercial systems, and only the trajectory log is used to model and analyze clone detection. The method can be applied to RFID systems in different frequency bands.
3. ACD has real-time performance. The algorithm analyzes trajectory logs in real time, and can detect clone tags in real time. Through experiments, it is found that the detection delay of 10,000 data points is only 3.273 s.

The remainder of this study is organized as follows: Section 2 introduces related research of RFID clone detection and the shortest path algorithm. Section 3 introduces the principle and process of ACD, and Section 4 discusses the implementation and method evaluation. Section 5 provides our conclusions. Section 4 describes the experiments, the results of which prove that the proposed approach significantly outperforms other existing approaches. Finally, a summary of the results and direction for future work are presented in Section 5.

## 2. Related Work

### 2.1. Trajectory-Based RFID Clone Detection

Trajectory-based RFID clone detection can be divided into probabilistic and deterministic according to detection results.

Probabilistic refers to the detection of anomalies by setting thresholds. We can obtain the characteristics of a normal trajectory by statistical learning or machine learning. The trajectory to be tested was compared with the normal trajectory characteristics, and the threshold will determine whether the trajectory was abnormal. Kamaludin et al. used statistical methods to identify abnormal tags [24]. A tag that frequently appears in one place is considered abnormal. Peichao et al. used the PrefixSpan algorithm to find frequent sequences of personnel behaviors [25] and determined abnormal sequences by a threshold. This method requires a huge dataset to learn characteristics, the detection accuracy is not high, and there are false negatives and false positives.

Deterministic refers to the tag path being certain and unique. A tag path that does not match the specified path is considered an exception [26]. Ouafi and Vaudenay [27] proposed the verification of tag authenticity by its compliance with a specified path. The protocol, Pathchecker, updates the tag state at each path step. At the end of a path, a reader knowing correct paths verifies whether the tag state reaches a proper value after updating by all readers on the correct path. The type has strong security requirements for tag state update. If a tag does not travel the correct path, then its state at the path end must not pass verification. It requires a predetermined correct trajectory, and it is not highly portable. All of the above detection methods require additional memory space to store dynamic trajectories, have higher memory read and write speed requirements, and increase the delay compared to other methods in communication between tags and devices.

Our proposed method has high detection accuracy, requires no additional memory space and does not need to know the correct trajectory. It is applicable to all of the current RFID systems.

### 2.2. Shortest Path Algorithm

The shortest path is a classic problem in graph theory. When there is more than one path between nodes in a graph, how do we find a path that minimizes the sum of the weights of the edges? Common algorithms applied to this problem are Dijkstra [28], Floyd-Warshall, Bellman-Ford, and SPFA. Dijkstra and Bellman-Ford are typical single-source shortest path algorithms and are often used to calculate the shortest path from one node to all other nodes. The Floyd–Warshall algorithm solves the shortest path problem between any two nodes and can be applied to directed graphs or negative weight graphs [29]. Its time complexity is high, but it can well solve the problem of cloning card detection, so we choose it for our application.

Researchers have applied the Floyd-Warshall algorithm to various fields and problems. Manaf et al. used it to determine the location of a field in a five-player match, helping find the shortest path to the opposite five-player team position [30]. Wolfram et al. applied it to detect closed-loop flow in power systems and solved the problem of the rapid increase in power flows faced by a transmission system [31]. Abdul and others applied it to optimize evacuation routes [32]. A good evacuation route allows one to more quickly evacuate from a place of disaster. We apply it to a new field, cloning tag intrusion detection in RFID systems.

## 3. Clone Tag Detection Algorithm

We introduce a clone tag detection algorithm based on trajectory data, using the feature of spatiotemporal collisions in spatiotemporal relations. Suppose that two nodes at a distance of 10 km read the same tag ID within one minute, but the tag could not pass both nodes in such a short time. This is called a spatiotemporal collision. We used the normal trajectories to construct the shortest time between two adjacent nodes, building a shortest time matrix between any nodes based on the Floyd-Warshall algorithm. Based on this, we proposed an online clone tag detection method.

### 3.1. Method Overview and Problem Definition

In the existing RFID system, using the spatiotemporal characteristics of the RFID data stream to detect clone tags is a fast and effective solution. Since RFID readers periodically collect tag data without interruption, RFID data streams have real-time, massive, and spatiotemporal correlation characteristics. In general, researchers use (tag ID, location, and time) triplets to express the collected RFID data information. tag ID represents the unique code of the tag. location is the location where the RFID reader reads the tag. time is the timestamp when the reading behavior occurs. The time of the same tag means that the same tag ID reflects the temporal relationship. The location reflects the changing process of the tag in space. In the network diagram, a location represents a detection node. The spatiotemporal association reflects the temporal and spatial connection of identified objects by tags.

**Figure 1:**
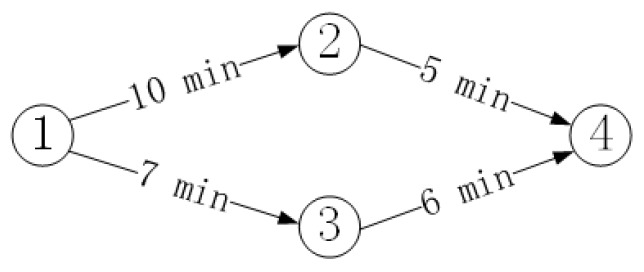
Network diagram.

We proposed ACD based on the spatiotemporal characteristics of the RFID data stream. This method uses the shortest path algorithm to acquire the spatiotemporal characteristics of the normal tags, and then compares the path to be measured with the normal path characteristics to identify abnormal tags. In the case of unchanged basic principles, to illustrate this method we simplified the actual complex road network diagram. As shown in Figure 1, {node_s→node_e, time} represents the time spent from node_s to node_e. According to this network diagram, the spatiotemporal characteristic that we built was {{node 1→node 2, 10 min},{node 2→node 4, 5 min},{node 1→node 3, 7 min},{node 1→node 4, 13 min}}. If {node 1→node 4, 5 min} appeared on a spatiotemporal relationship, we thought it was abnormal. In addition, there might be a cloning attack in the system, because 5 min is obviously less than 13 min.

**Figure 2:**
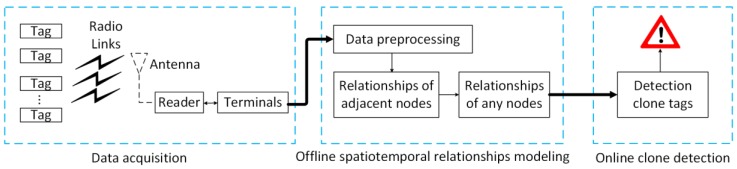
Flowchart of the detection method.

ACD mainly includes two stages: offline and online, as shown in Figure 2. The data preprocessing process refers to integrating the original data stream obtained in the system into a trajectory according to the tag ID. Relationships of adjacent nodes refer to intuitively calculating the time interval between two adjacent nodes in the trajectory. Relationship of any nodes refers to calculating the time interval between all nodes in the system, and constructing the spatiotemporal characteristics of the normal trajectory. The online detection stage refers to integrating the real-time data stream in the system into trajectory data, and then comparing with the normal trajectory to detect an abnormal trajectory.

### 3.2. Data Structure Definition

We used the log from a file tracking system based on UHF RFID to track file transfer trajectories. As shown in Figure 1, the RFID system includes tags, antennas, readers, and terminals [33]. One reader can connect several antennas. An antenna is uniquely identified by the reader port number. One antenna can uniquely determine a location, referred to as a node. The tag is attached to the identified object. When that object enters the reading range of the antenna, the tag and antenna transmit information through spatial electromagnetic coupling. The tag sends information to the reader, which decodes it and sends it to the terminal processing system in real time, so as to identify the object.

The log generated in the terminal system included a node information table and node record table. The node information table stored the node ID, reader ID (IP), reader port number, and node location description. The node record table recorded when the tag passes by a node, and included the tag ID, recording time, and node ID, as shown in Table 1. The node information table was updated less frequently, and the node record table was continuously updated with the transfer of tags. We only used Table 1 to detect a clone tag.

Assume there are N labels and M nodes in an RFID system. T is the tag ID. The i-th tag is $T_{i}\left(i=1,2\ldots N\right)$. $T_{i}^{c}$ is the cloned tag of $T_{i}$. Time is represented by t, and the position of tag $T_{i}$ at time $t_{j}$ is $L_{j}^{i}$. $\left\{L_{1}^{i},\;L_{2}^{i},\ldots \;,L_{j}^{i}\right\}$ is the trajectory sequence of $T_{i}$. For example, in Table 1, $T_{i}=F01000310F30010011712011$, and its trajectory sequence is {002,003,001,004}.

### 3.3. Data Preprocessing

Most commercial devices have middleware for data cleaning, so we assumed that data exported from a terminal system have no redundancy and can be processed directly as input data for clone detection.

The node record table recorded when and where tags passed; the data were grouped by tag ID and were sorted by time. Then, the trajectory sequence was formed. It subtracted adjacent times and returned the result. The result was the time for the tag to pass between adjacent nodes.

### 3.4. Offline Spatiotemporal Relationship Modeling

In this stage, we first obtained the spatiotemporal relationship between the neighbor nodes by statistical methods, and then obtained the spatiotemporal relationship between any nodes based on the shortest path algorithm. The shortest path algorithm is a set of algorithms aimed at finding a path that minimizes the sum of the weights of the edges when there is more than one path between nodes in a graph. Common algorithms applied to this problem are Dijkstra, Floyd-Warshall, Bellman-Ford, and SPFA. Dijkstra and Bellman-Ford are typical single-source shortest path algorithms and are often used to calculate the shortest path from one node to all other nodes. The Floyd-Warshall algorithm solves the shortest path problem between any two nodes. It is suitable for cases in which the starting point and ending point are not fixed. Therefore, in this study the Floyd-Warshall algorithm was selected to calculate the shortest arrival time.

#### 3.4.1. Relationships of Adjacent Nodes

We could obtain several time intervals through data preprocessing. We assumed there were no clone attacks, so the obtained time intervals were reasonable. By observing the data, it could be concluded that the time interval between two points was uniformly distributed. From these distributions, we could calculate the minimum reasonable time for a tag to traverse a specified path. We used $t_{ab}$ to identify the shortest arrival time from node a to node b. We synthesized all nodes to obtain the shortest time matrix between adjacent nodes:



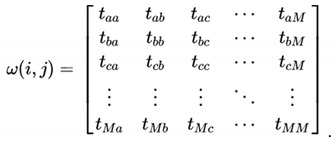



#### 3.4.2. Relationships of Nodes

After obtaining the shortest time between adjacent nodes, we used the Floyd-Warshall algorithm to calculate the shortest times between all pairs of nodes [34]. Floyd-Warshall uses dynamic programming to find the shortest path between multiple source points in a given weighted graph. It can be used for any graph, including directed graphs and graphs with negative weighted edges. Starting from the first point, each point in turn acts as an intermediate k. For each pair of points i and  j, we checked whether there was a path through k that is shorter than the known path, and updated it if such a path exists. Algorithm 1 is the pseudocode of the Floyd-Warshall algorithm. The output of the algorithm is dis, which is the shortest time matrix of the input network graph.
**Algorithm 1** The Floyd-Warshall’s shortest path algorithm**Input:**   The initial weights of the graph: w(i,j)   The number of vertices: n**Output:**   The final matrix of the shortest paths: dis(i,j)1: **for**
i=1 to n
**do**2:   **for**
j=1 to n
**do**3:     dis(i,j)=w(i,j);4:   **end for**5: **end for**6: **for**
k=1  to n
**do**7:   **for**
i=1 to n
**do**8:     **for**
j=1 to n
**do**9:      dis(i,j)=min(dis(i,j),dis(i,k)+dis(k,j));10:     **end for**11:   **end for**12: **end for**13: return dis;

The network graph formed by the trajectory is bidirectional, which means that the shortest time matrix is symmetric. We improved the classic Floyd-Warshall algorithm based on the characteristics of a symmetric matrix, and our results show that it could effectively reduce the running time. Algorithm 2 is the improved Floyd-Warshall algorithm, through which we could obtain the spatiotemporal relationship matrix between adjacent nodes and perform online clone detection.
**Algorithm 2** The improved Floyd-Warshall’s shortest path algorithm**Input:**   The initial weights of the graph: w(i,j)   The number of vertices: n**Output:**   The final matrix of the shortest paths: dis(i,j)1: **for**
i=1 to n
**do**2:   **for**
j=1 to n
**do**3:     dis(i,j)=w(i,j);4:   **end for**5: **end for**6: **for**
k=1  to n
**do**7:   **for**
i=1 to n
**do**8:     t=dis(i,k)9:      **for**
j=1 to i
**do**10:      dis(i,j)=min(dis(i,j),t+dis(k,j));11:      dis(j,i)=dis(i,j);12:      **end for**13:   **end for**14: **end for**15: return dis;

### 3.5. Online Clone Detection

Comparing real-time data with the data of the shortest time matrix dis, we could detect the clone tag. In logs collected in real time, when the time interval between two adjacent records with the same ID was less than that in dis, the system considered that there was an exception, and an alarm was triggered. The administrator will know the locations and ID of the cloning tag. For example, assume the genuine tag $T_{i}$ passes point $L_{j}^{i}$ at $t_{j}$, and $T_{i}^{c}$ passes $L_{k}^{i}$ at $t_{k}$. The anomaly detection rule is:

$$status=\{\begin{array}{l} abnormal\;if\;t_{k}-t_{j}<dis\left(L_{j}^{i},\;L_{k}^{i}\right)\;\\ normal\;otherwise. \end{array}$$

**Figure 3:**
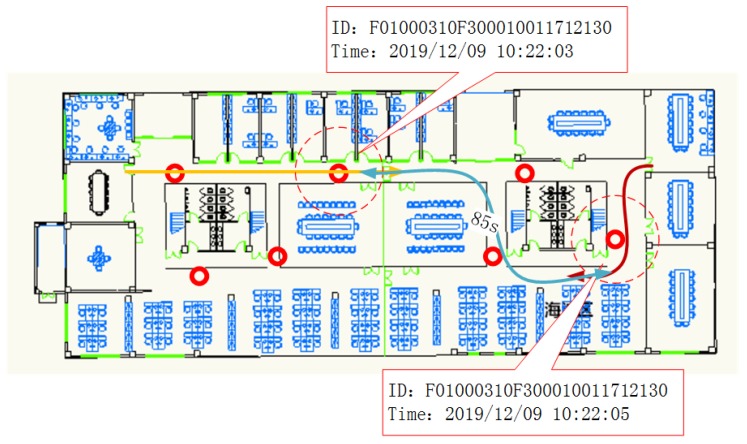
Abnormal detection.

When an abnormal tag is detected, the system displays the abnormal location. Administrators can pinpoint the locations of anomalies and view anomalous labels on the spot. As shown in Figure 3, the alarm MessageBox displays the ID and reading time of the tag. There are two tags with the same ID, and their time interval is 2 s. The yellow line indicates the true tag trajectory, the red line indicates the cloned tag trajectory, and the blue line indicates the shortest path between two nodes, with the shortest time being 85 s. Obviously, 2 s is less than 85 s, so the system detects an abnormal event and triggers an alarm.

## 4. Implementation and Evaluation

### 4.1. Experiment Deployment

**Figure 4:**
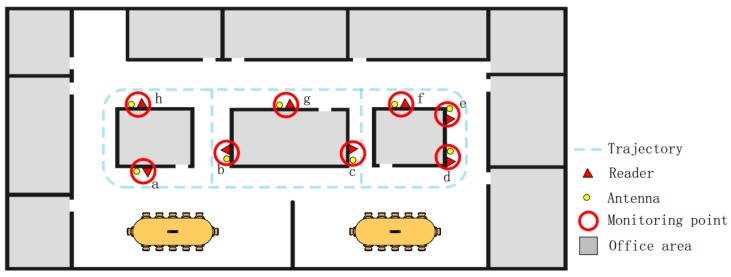
Vertical view of experimental area.

**Figure 5:**
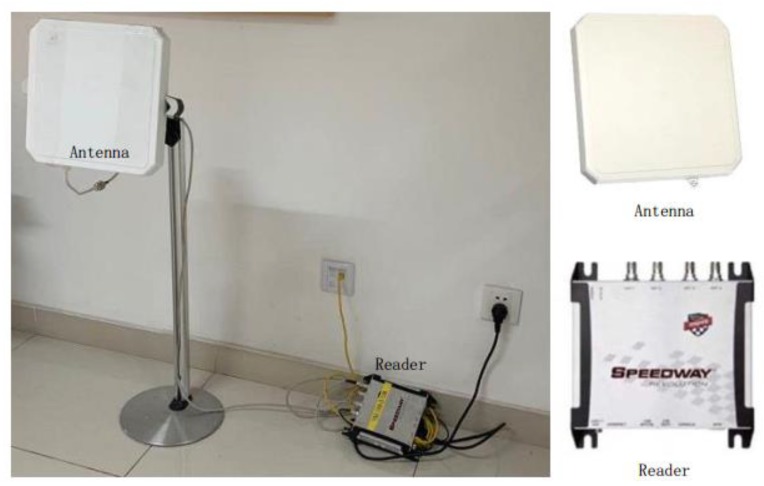
System deployment.

We used Impinj Speedway R420 readers, Larid S9028 antennas, and EPC tags. These are all COTS RFID devices. RFID tags were pasted on the A4 paper, which was six pages thick. We tested in an office area of approximately 500 m^2^. The specific deployment method is shown in Figure 4. To obtain the influence of the number of nodes on the detection accuracy, we designed five scenarios. We deployed four, five, six, seven, and eight nodes for data acquisition, where the distance between the nodes a, b, c, d, f, g, and h was large, and the communication between the antenna and the tag in adjacent nodes would not interfere with each other. Node e was close to f and d. When the tag was between nodes e and f, it might communicate with the antenna in both nodes. Except for the number of nodes, the number of genuine and clone tags and the experimental process were the same. We selected eight nodes of the experimental process for detailed introduction. Each node was placed with an antenna and reader. The specific deployment method is shown in Figure 5. To optimize the communication of the antenna and tag, we set the height of the antenna to be approximately 1 m, which was approximately the same as the height of the tag held in the hand when passing the antenna. During the experiment, we connected eight detection nodes to the server through the network for unified control and data acquisition. The power modulation of the reader and antenna was the maximum, and the volunteers walked normally in accordance with the blue route.

In this experiment, we defined the trajectory of the volunteers. The volunteers were able to walk freely along the blue dotted line. In different scenarios, we selected 1000 tags as genuine tags, and selected 10 volunteers to enter the experimental area with genuine tags in batches, so as to obtain the normal spatiotemporal relationship between nodes. Then, 100 of them were selected and cloned. The 10 volunteers were divided into two teams: A and B. A took the genuine tags and B took the clone tags, and ensured that clone tags and corresponding genuine tags entered the experimental area within the same time period. The Table 2 shows the node numbers selected in different scenarios and the size of the datasets collected.

### 4.2. Clone Detection

#### 4.2.1. Detection Accuracy of the Clone Event

Precision and recall are the most common indicators to judge the quality of a classification model. Precision is the percentage of samples judged to be positive. Recall is the percentage of all correctly detection results to all should be selected results. We used the cumulative distribution function (CDF) to evaluate the detection accuracy of this method over a long period of time.

**Figure 6:**
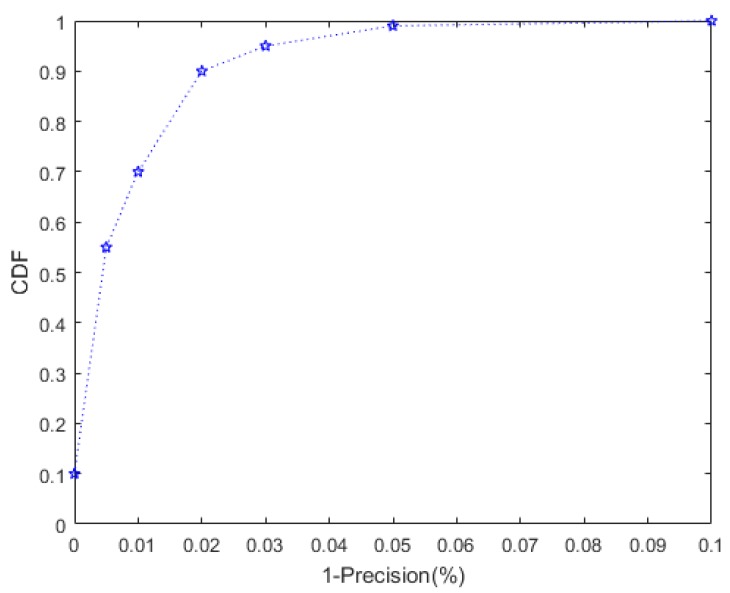
Detection accuracy of the clone event.

We plotted the CDF in Figure 6. In most cases, the detection precision could reach 98%. Sometimes the accuracy could reach 100%. It should be noted that under the proper deployment distance, increasing the number of antennas could improve the accuracy.

**Figure 7:**
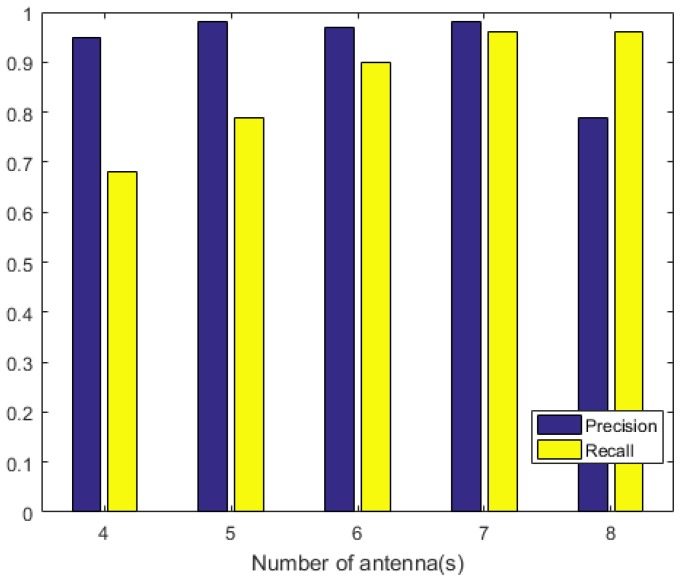
Impact of the number of antennas on the precision and recall.

We now evaluated the impact of the number of antennas on precision and recall in this experimental scenario. Our results are shown in Figure 7. It can be seen that when the number of antennas was 4–7, the precision and recall gradually approached 100%. However, when the number of antennas was 8, there was a significant decrease. This was inconsistent with theory. By analyzing the position of the antenna deployed in the experimental environment, the original collected data, and the results, we found that the distance between nodes was small. Normal tags were easily mistaken for abnormalities, which affected accuracy. Therefore, when ACD is used, we must avoid placing antennas close to each other, and should arrange an appropriate number of antennas according to the experimental scenario.

#### 4.2.2. Real-Time Detection of Clone Event

**Figure 8:**
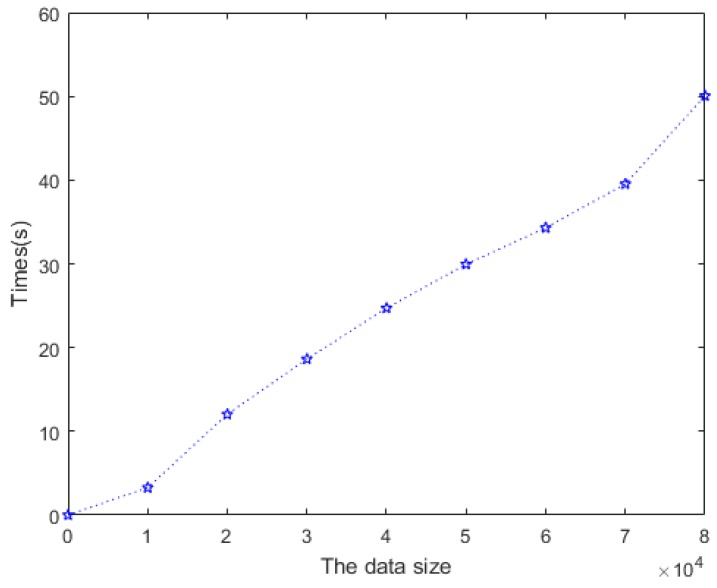
Delay of the cloning detection method.

In this experiment, we deployed thousands of tags attached to people. The data collected by the background system increased with the number of tags and the mobility. The online detection model will be congested, a large amount of data will appear in the buffer pool, and there will be a delay in the detection model. We evaluated the processing latency of ACD for different data volumes. Figure 8 shows the ACD time delay for different dataset sizes. Obviously, the delay was proportional to the amount of data. If there are 10,000 pieces of data in the buffer pool, then the online detection time of the model is delayed by 3.273 s. This means that ACD is real-time, capable of processing large amounts of data, and can be applied to scenarios with a large number of tags and strong mobility.

#### 4.2.3. Effectiveness of the Clone Method

**Figure 9:**
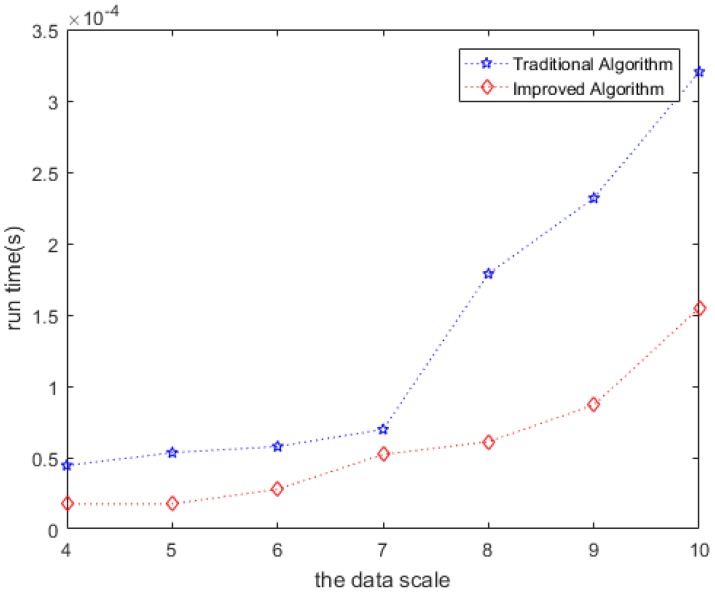
Comparison of the runtime between classic and improved algorithms.

We improved the classic Floyd-Warshall algorithm for offline trajectory modeling. The experimental results show that the improved algorithm has a shorter running time and higher efficiency under the same data scale. In Figure 9, the horizontal axis represents the number of nodes M, and the vertical axis is the algorithm running time. The blue line represents the improved algorithm, and the red line represents the traditional algorithm. Obviously, the improved algorithm has a shorter running time, and the effect becomes greater with the number of nodes.

### 4.3. Adaptivity of the Proposed Scheme

**Figure 10:**
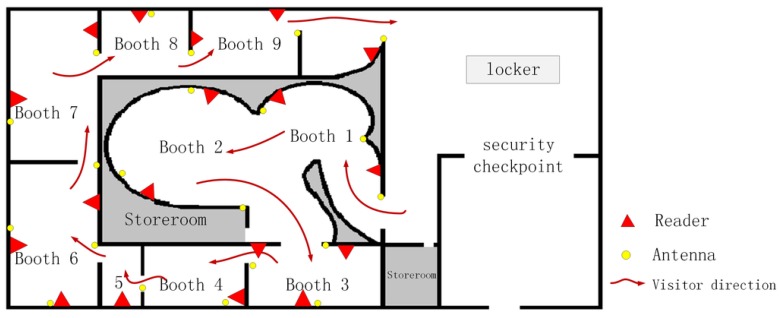
Vertical view of the large venue.

To verify the adaptivity of this method, we used the dataset in the product expo for experiments. The dataset was generated in a venue covering approximately 6000 m^2^. The specific structure of the venue is shown in Figure 10. According to the layout characteristics of the venue, we deployed a total of 18 readers and 21 antennas. The reading and writing cycle of the reader was 5 s, which means one tag was read every 5 s. To ensure the order of the venue, the organizer stipulated that the audience could only visit in one order. The dataset contained more than 400,000 pieces of data and was formed by approximately 10,000 tags. The duration of the exhibition was short, number of visitors large, and population density of the booth large, so the communication between the reader and the tag was sometimes hindered. The characteristic reflected in the data was that there were numerous missed readings. Statistics found that only about 35% of the data was relatively complete. We used this dataset as the training set. Then, we added 100 trajectories to the original dataset, and these trajectories represent the trajectories generated by 100 clone tags. They simulate the characteristics of the real trajectory, and the stay time in the exhibition hall was the same as the normal visit time, approximately 1 h. Among them, 65 tracks had the feature of missed reading and 35 tracks were relatively complete. We used the newly generated dataset as the detection dataset.

**Figure 11:**
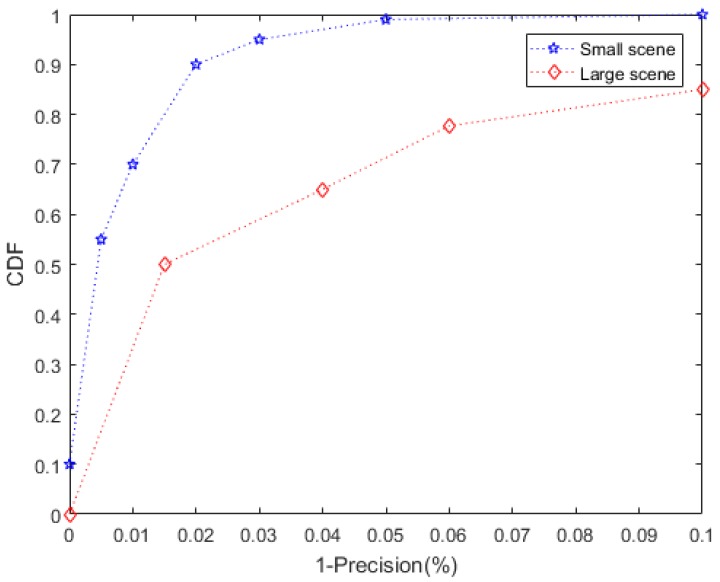
Precision of clone detection in a large area.

We used CDF for evaluation, and the results are shown in Figure 11. The precision and recall under this dataset decreased significantly. We analyzed that the reason for this result was the large number of people in the venues, the high density, and low number of readers. These increase the false alarm and false alarm rate of the reader. People did not follow the regulations for one-way visits and moved freely in the venue. Although the precision and recall were reduced, it still had a good effect. Therefore, this method could be adapted to venues of different sizes, and only needs tags in venues to flow very frequently.

### 4.4. Discussion

The experimental results show that our proposed method has certain advantages in terms of accuracy, real-time performance, short communication delay, adaptability, and use of only COTS devices. At present, there are many methods for detection of RFID cloning attacks. We selected some detection methods similar to ACD for comparison from deterministic, probabilistic, COTS devices, but not adding communication delay and real-time.

For example, the Pathchecker proposed by Ouafi and Vaudenay advocates verifying tag authenticity using its compliance with the specified path [27]. Each time the tag passes through a reader, the path recorded in the memory is updated. At the end of a path, a reader knowing correct paths verify whether tag state reaches the value as it should do after being updated by all readers on the correct path. The detection accuracy of this method is high. However, it needs to set a hash function in the tag to encrypt each path update, requires the memory of tags to store the actual path, and requires readers to store the correct path. The update of the path in the tag will increase the communication time between the tag and the reader. Detection at the end of the path through path comparison makes the detection not real-time. Same as Pathchecke, Tracker also requires a centralized manager/server for path verification [19]. Maleki et al. proposed Lightsource, which introduces non-volatile memory (NVM) on a tag to store obfuscated tag trace [21]. Obviously, Lightsource needs to update the COTS equipment. Elkhiyaoui et al. propose CHECKER to enable any reader on a path to check whether a tag has through a valid path so far. To achieve this, CHECKER requires that each reader stores all valid paths leading to it [35].

In conclusion, ACD has more advantages than other trajectory-based cloning detection methods. The specific comparison results are shown in Table 3.

## 5. Conclusions

This study proposed ACD, an effective method to detect clone tags using the COTS RFID equipment and the Floyd-Warshall shortest path algorithm based on spatiotemporal collisions. Experimental results show that the precision of our clone attack detection could reach 100% in a short time, the precision could reach 98.7%, and the recall rate could reach 96% over a long time. ACD can detect clone tags in real time. Administrators can quickly locate abnormal locations through visual alarm displays. This method has a good tolerance for data incompleteness. ACD can be adapted to most scenarios in which RFID tags are scanned frequently. Such as RFID-based ticket security detection, personnel authentication, material supply chain management, personnel management in large exhibition, etc. This method is a lightweight detection method without professional equipment. It can solve some of the harm caused by cloning attacks, such as traceback of counterfeit materials in the supply chain, access of non-compliant personnel to controlled areas. Additionally, it improves the security of RFID sensors and application systems.

In the future, we plan to track the technology of PUFs and try to combine our scheme with this technology to better improve the security of RFID systems. We also plan to apply this method to detection effects in actual applications.

## Figures and Tables

**Table 1:** Node record table.

Tag ID.	Node ID	Recording Time
00B07A13E010A24800000266	003	2019/12/4 14:16:07
00B07A13E010A24800000266	001	2019/12/4 14:06:23
F01000310F30010011712011	003	2019/12/4 14:07:34
F01000310F30010011712011	002	2019/12/4 14:04:12
00B07A13E010A24800000266	002	2019/12/4 14:10:12
F01000310F30010011712011	004	2019/12/4 14:40:28
F01000310F30010011712011	001	2019/12/4 14:25:00
…	…	…

**Table 2:** Data set introduction.

Number of Nodes	Node Sequence Number	Dataset Size (Normal Datasets\Test Set)
4	a, d, g, h	34,076\36,034
5	a, c, d, g, h	45,043\53,724
6	a, c, d, f, g, h	50,419\57,812
7	a, b, c, d, f, g, h	63,032\68,102
8	a, b, c, d, e, f, g, h	78,939\90,176

**Table 3:** Comparison of clone detection methods.

Name of Methods	Deterministic	Probabilistic	COTS	Not Add Communication Delay	Real-Time
Pathchecker	√		√		
Tracker	√		√		
Lightsource	√				√
CHECKER		√		√	√
ACD	√		√	√	√
